# Antibiotic sensitivity in correlation to the origin of secondary peritonitis: a single center analysis

**DOI:** 10.1038/s41598-020-73356-x

**Published:** 2020-10-29

**Authors:** Rainer Grotelüschen, Lena M. Heidelmann, Marc Lütgehetmann, Nathaniel Melling, Matthias Reeh, Tarik Ghadban, Anna Dupree, Jakob R. Izbicki, Kai A. Bachmann

**Affiliations:** 1 Department of General, Visceral and Thoracic Surgery, University Medical Center Hamburg-Eppendorf, Martinistrasse 52, 20246 Hamburg, Germany; 2 Institute for Medical Microbiology, Virology and Hygiene, Medical Center Hamburg-Eppendorf, 20246 Hamburg, Germany

**Keywords:** Bacterial infection, Gastrointestinal diseases

## Abstract

Despite improvements in diagnosis, intensive-care medicine and surgical technique, the mortality of patients with secondary peritonitis is still high. Early and aggressive empiric antibiotic treatment has strong impact on the outcome. This retrospective study investigates bacterial and fungal pathogens and their antibiotic sensitivity in patients with secondary peritonitis. All patients that underwent emergency laparotomy due to secondary peritonitis at the Department of Surgery, University Medical Center Hamburg-Eppendorf between 2005 and 2015 were reviewed and overall 414 patients were included. We correlated the intra-abdominal localization of the organ perforation with intraoperative microbiological findings and corresponding sensitivities to relevant antibiotics. Overall, the most common findings were *Escherichia coli* (39%) and other Enterobacterica (24%). Depending on the location of the perforation, Cefuroxime/Metronidazole and Cefutaxime/Metronidazole were effective (based on in vitro susceptibility testing) in only 55–73% of the patients, while Meropenem/Vancomycin was able to control the peritonitis in more than 98% of the patients; independent of the location. Besides early source control, appropriate empiric treatment plays a pivotal role in treatment of secondary peritonitis. We are able to show that the frequently used combinations of second or third generation Cephalosporins with Metronidazole are not always sufficient, which is due to the biological resistance of the bacteria. Further clinical studies are needed to determine whether calculated use of broad-spectrum antibiotics with a sensitivity rate > 99%, such as Carbapenem plus Vancomycin, can improve overall survival rates in critically ill patients with secondary peritonitis.

## Introduction

Peritonitis can be divided into three subtypes: primary, secondary and tertiary peritonitis^1^. This study focuses on secondary peritonitis, which is an inflammatory reaction in the abdominal cavity caused by perforation of hollow organs and in many cases leads to severe sepsis with organ failure.

The mortality rate in patients with sepsis is 15–25% and can be as high as 18–55%^2^ when gram-positive Cocci are present. These are also associated with a higher rate of early deaths^2,3^. Peritonitis is the cause of sepsis in 5–70%. Overall, sepsis due to peritonitis is associated with a severe course of the disease resulting in increased sepsis severity scores^4,5^.

Origin of the peritonitis and effects of antimicrobial treatment are the main factors influencing the severity of peritonitis and its outcome. Mortality and morbidity of sepsis or severe peritonitis can be reduced by state of the art critical care medicine, including fluid resuscitation, vasopressor therapy and surgical or interventional source control.

It has been shown that early empiric antibiotic treatment and surgical source control can reduce mortality^4,6^. Improved intensive care and surgical management as well as more targeted diagnostics of peritonitis have reduced mortality from 90% in 1900 to 15–25% (9, 11). However, due to increasing microbial resistance, appropriate antibiotic treatment is getting more and more challenging, especially empiric treatment. Ruettinger et al. (et alii) found unsuitable administration of antibiotics in 30% of their cases with secondary peritonitis^5^.

Commonly, the empiric treatment of secondary peritonitis includes a combination of antibiotics, such as second or third generation Cephalosporins (Cefuroxime/Ceftriaxone), plus Metronidazole or Piperacillin/Sulbactam^7^. In patients with severe sepsis, broad-spectrum antibiotics, such as Meropenem are frequently used^8,9^.

Several studies concerning appropriate antibiotic treatment in patients with sepsis or peritonitis have previously been published^10,11^. However, there is only little data available analyzing the origin of peritonitis. Therefore, the aim of this trial is to investigate the intraoperative microbial findings and their sensitivity to antibiotic treatment so as to evaluate the present empiric treatment strategies in secondary peritonitis. Additionally, the impact of the origin of the peritonitis in regard to the detected bacteria, the effective antibiotics and their impact on mortality was analyzed.

## Material and methods

### Study design and patients

The study included 414 consecutive patients, which underwent emergency surgical therapy for secondary peritonitis at the Department of Surgery at the University Medical Center Hamburg-Eppendorf between 2005 and 2015. The data were retrospectively retrieved from our prospective database. The trial was approved by our institutional review board. The University Medical Center Hamburg Institutional Review board belongs to University medical Center Hamburg. According to local laws, no informed patient consent or statement by the federal ethics committee is needed since the study is non interventional and retrospective (§12HmbKHG—city law Hamburg).

Patients with secondary peritonitis were identified by evaluating the surgical emergencies in the defined period cross checked with an ICD-10 (International Classification of Diseases-10) search for “hollow organ perforation” and “peritonitis”. A total of 2398 patients were screened. Patients with primary peritonitis or postoperative peritonitis were excluded, as these do not represent secondary peritonitis. In patients that required more than one operation, only the results from the first procedure were included in the analysis. The patients were grouped into five categories according to the origin of the peritonitis (colon, stomach, duodenum, small intestine and biliopancreatic). The origin was mainly due to perforation of the respective organ. During the procedures microbiological cultures were taken. All patients underwent adequate surgical source control, which ranged from simple suturing of the perforation site to discontinuous organ resection. The indication for staged lavage depended on the surgeon’s evaluation of the initial intraoperative findings.

### Clinicopathological data

Data including patients’ sex, age, date of the operation, medical history, medication, comorbidities, microbial findings and sensitivity, origin of the peritonitis and mortality were obtained from our prospective database and the respective clinical records.

### Microbiological samples and antibiotic treatment

Intraoperative microbiological sampling was performed as a routine of all surgical interventions in patients with secondary peritonitis. Specimens (peritoneal fluid/tissue) were collected in every primary and redo operation from the site of infection.

Susceptibility results were retrospectively retrieved from the patient chart. The testing of all microbiological samples was performed at the Department of Microbiology and Virology, University Medical Center Hamburg-Eppendorf, according to standardized protocols using selective and non-selective agar plates. The sensitivity was analyzed^9^ by agar diffusion or VITEK® analyzer (Biomerieux, France). Results of sensitivity testing were standardized according to the EUCAST (European Committee on Antimicrobial Susceptibility Testing) and German guidelines.

If not tested separately, in case of distinct knowledge of sensitivity based on other tested antibiotics of the microorganism, the result was determined according to the “47th edition of The Sanford Guide To Antimicrobial Therapy”^12^. The antibiotic treatment was adjusted when the septic situation persisted or when inflammation parameters did not decrease as expected. Furthermore, antibiotic administration was deescalated according to the results of the intraoperative smears.

The overall rate of sensitivity for the Antibiotics was calculated by adding up the results of the columns adjusted by the absolute number in the column.

The presence of microbes and their antibiotic resistances were grouped as follows: *Enterococcus faecium*, *Enterococcus faecalis*, other Enterococcus species, *Escherichia coli*, other Enterobactericae (non *E. coli*), Staphylococci, Streptococci, Bacteroides species, Yeasts, gram-positive bacteria and gram-negative bacteria.

The rate was calculated by diverting the number of sensitive bacteria (against the mentioned antibiotic) by the total number of the bacteria for each kind of bacteria and overall.

### Mortality

The mortality rate was calculated for the various origins of the secondary peritonitis and for the different bacteria. Additionally, the impact of antibiotic resistance on the mortality rate was analyzed.

### Statistics

Data were analyzed using SPSS® for Windows® (22.0; SPSS Inc., Chicago, IL) and reported in descriptive charts. Cross-tables were generated, *p* values were calculated with the chi-squared test/Fisher’s exact test. Significance refers to *p* values for two-tailed tests of less than 0.05. Multivariate analysis was performed using the multiple logistic regression analysis.

## Results

In this trial, 414 patients that underwent emergency laparotomy due to secondary peritonitis at the University Medical Center Hamburg Eppendorf, were included. Most frequently, the source was located in the colon (56% of the patients) followed by stomach (15%), biliopancreatic system (12%), duodenum (10%) and small intestine (7%) (Table 1).

**Table 1:** Clinical data.

	Patients		Mortality (%)	*p* value
Overall mortality	(66/414)		16	
**Age**				
Age ≤ 62	(n = 208)	50%	8	**< 0.001**
Age > 62	(n = 206)	50%	25	
**Sex**				
Male	(n = 228)	55%	15	0.213
Female	(n = 186)	45%	19	
**Location**				
Colon	(n = 234)	56%	15	**0.012**
Stomach	(n = 61)	15%	15	
Duodenum	(n = 40)	10%	28	
Small Intenstine	(n = 28)	7%	14	
Pancreas/biliary tract	(n = 51)	12%	12	
**ASA score**				
ASA I	(n = 21)	5%	5	** < 0.001**
ASA II	(n = 145)	35%	5	
ASA III	(n = 178)	43%	15	
ASA IV	(n = 70)	17%	46	
**Preexisiting condition**				
Cirrhosis	(n = 28)	7%	23 versus 15	0.183
Diabetes	(n = 60)	14%	29 versus 14	**0.001**
Chronic heart disease	(n = 101)	24%	32 versus 13	**< 0.001**
Immunosuppression	(n = 42)	10%	18 versus 15	0.575

Bold value indicates *p* < 0.05.

### Clinicopathological data

Mean age of the patients was 62 (18–95) years; 55% were male and 45% female. The median SOFA score (Sepsis-related organ failure assessment score) was 2 (0–16). The median CRP (C-reactive protein) value of the patients was 125 mg/l (5–535) and the median leucocyte count was 14 × 10^9^/l (1–55). Length of postoperative ICU (Intensive Care Unit) stay and hospital stay was 5 days (0–93) and 14 days (1–373), respectively (Table 1). Mortality was found to be 16%, with ASA score (American Society of Anesthesiologists score), location of origin of the peritonitis, preexisting chronic heart disease and diabetes identified as prognostic parameters in univariate analysis. In multivariate regression analysis, a high SOFA score was identified as the sole independent prognostic factor for mortality (Table 2).

**Table 2:** Multivariate analysis.

Parameter	OR	95%CI	*p*
Age	1.115	0.525–2.368	0.777
Sex	1.402	0.741–2.651	0.299
Location	0.918	0.773–1.091	0.331
SOFA score	29.033	14.081–59.862	
ASA score	1.657	0.678–4.051	0.268
Cirrhosis	0.675	0.231–1.967	0.471
Diabetes	1.277	0.575–2.837	0.548
Chronic heart disease	1.388	0.67–2.767	0.351
Immunosuppression	0.749	0.312–1.798	0.518

### Microbial flora and location

Overall 589 pathogens were detected in the 414 patients. The most common findings were *E. coli* (39%) and other Enterobactericae (24%), followed by Yeast (22%). Bacteriodaceae were found in 22%, followed by Enterococcus species in 20% of the swabs. 20% of the samples were sterile (Table 3). Analyzing the distribution of the bacteria, relevant differences in respect to the different localizations were detected.

**Table 3:** Microbial flora related to location.

	Colon	Stomach	Duodenum	Small intestine	Pancreas/biliary tract	Overall
n =	234	61	40	28	51	414
Enterococcus, not specified	10%	6%	12%	12%	11%	10%
*Enterococcus faecium*	8%	2%	6%	4%	5%	6%
*Enterococcus faecalis*	3%	7%	9%	0%	5%	4%
*Escherichia coli*	52%	9%	9%	50%	36%	39%
Enterobactericae	26%	6%	21%	46%	24%	24%
Staphylococcus	7%	11%	15%	12%	9%	9%
Streptococcus	7%	11%	15%	4%	9%	8%
Bacteroidaceae	31%	4%	6%	19%	20%	22%
Other gram positive	5%	2%	0%	8%	5%	4%
Other gram negative	11%	0%	6%	4%	7%	8%
Yeast	15%	43%	33%	23%	23%	22%
Sterile	13%	43%	30%	15%	22%	20%

In patients with colonic perforation, *E. coli* was detected in more than 50%. In contrast, when reviewing patients with stomach perforation, sterile swabs were detected in 43% of the patients; *E. coli* and other Enterobacteriacae were found in 9% and 6% only (Table 3).

### Biological resistance and microbial flora

The sensitivity rate for the most commonly used antibiotics and their common combinations were determined in relation to the detected microbes. In the microbial cultures, the sensitivity rate for Piperacillin/Sulbactam was 82%, overall.

The best rates were found for *E. faecalis* (100%), Staphylococcus (97%), Streptococcus (96%) and other gram-positive bacteria (91%).

Administration of Meropenem lead to excellent rates for *E. coli*, other Enterobacteriacae, Streptococcus and Bacteriodaceae (all 100%), Staphylococcus (96%) and other gram-positive bacteria (86%). The overall sensitivity rate was 78%.

Compared to this, the frequently used combination of Cefuroxime or Cefotaxime plus Metronidazole, showed far lower rates of sensitivity (65% and 69%, respectively). These combinations only provided good results for *E. coli*, Streptococcus and for Bacteroidaceae. The best antimicrobial coverage was found for the combination of Meropenem with Vancomycin revealing an overall sensitivity rate of 98% (Table 4).

**Table 4:** Biological sensitivity related to microbial flora.

	Enterococcus, not specified	*Enterococcus faecium*	*Enterococcus faecalis*	*Escherichia coli*	Enterobactericae	Staphylococcus	Streptococcus	Bacteroidaceae	Other gram positive	Other gram negative	Overall
N =	41	25	16	162	99	37	34	92	17	32	555
Ampicillin/ Sulbactam	69%	0%	100%	73%	47%	61%	96%	87%	5%	66%	66%
Piperacillin/ Sulbactam	69%	0%	100%	78%	87%	97%	96%	87%	91%	81%	82%
Meropenem	0%	0%	0%	100%	100%	96%	100%	100%	86%	83%	78%
Cefuroxime	0%	0%	0%	96%	55%	64%	96%	99%	5%	57%	57%
Cefotaxime	0%	0%	0%	97%	91%	21%	100%	40%	10%	62%	61%
Ceftazidime	0%	0%	0%	97%	90%	4%	15%	40%	95%	59%	56%
Tigecycline	97%	100%	100%	99%	75%	100%	100%	82%	5%	87%	88%
Ciprofloxacin	0%	0%	0%	90%	99%	75%	4%	0%	95%	55%	53%
Moxifloxacin	91%	0%	21%	60%	99%	96%	96%	43%	5%	67%	68%
Vancomycin	100%	88%	100%	4%	0%	100%	100%	0%	0%	33%	36%
Metronidazole	0%	0%	0%	0%	0%	0%	0%	97%	0%	17%	16%
Cefuroxime/Metronidazole	0%	0%	0%	96%	55%	64%	96%	97%	5%	66%	65%
Cefotaxime/Metronidazole	0%	0%	0%	97%	91%	21%	100%	97%	10%	70%	69%
Meropenem/Vancomycin	100%	88%	100%	100%	100%	100%	100%	100%	86%	99%	98%

### Biological resistance and location of the perforation

We evaluated frequently used antibiotics in respect to their sensitivity rates in empiric treatment of the different origins of peritonitis. Empiric treatment with Piperacillin/Sulbactam was effective, particularly in patients with perforations of the stomach and duodenum (90 and 91%), while the rates for perforations of the colon and small intestine were 78% and 83% respectively. Meropenem was found to be less effective in the upper gastrointestinal tract, with a sensitivity rate in stomach and duodenal perforations of 74% and 70%, respectively, than in colonic perforations (87%). The combination of Cefuroxime or Cefotaxime plus Metronidazole only showed sensitivity rates ranging between 55 and 73%, which was lower than Piperacillin’s sensitivity rates independent of the localization. The results of Cefotaxime plus Metronidazole were slightly better than those of Cefuroxime plus Metronidazole (69 vs. 65% overall). The combination of Meropenem plus Vancomycin was found to be the most effective treatment for all localizations with sensitivity rates of 98% overall. Details are shown in Table 5.

**Table 5:** Biological sensitivity related to location.

	Colon	Stomach	Duodenum	Small intestine	Pancreas/ biliary tract	Overall
	234	61	40	28	51	414
Ampicillin/Sulbactam	63%	74%	73%	67%	66%	66%
Piperacillin/Sulbactam	78%	90%	91%	83%	81%	82%
Meropenem	87%	74%	70%	71%	53%	78%
Cefuroxime	55%	65%	58%	52%	57%	57%
Cefotaxime	62%	55%	64%	62%	62%	61%
Ceftazidime	63%	36%	46%	56%	59%	56%
Tigecycline	83%	97%	97%	94%	87%	88%
Ciprofloxacin	56%	45%	52%	48%	55%	53%
Moxifloxacin	61%	81%	82%	73%	67%	68%
Vancomycin	24%	65%	58%	46%	33%	36%
Metronidazole	21%	7%	6%	10%	17%	16%
Cefuroxime/Metronidazole	67%	65%	58%	56%	66%	65%
Cefotaxime/Metronidazole	73%	55%	64%	65%	70%	69%
Meropenem/Vancomycin	99%	97%	97%	98%	99%	98%

### Mortality

66 out of 414 patients died, resulting in an in-hospital mortality rate of 16% (Table 1). The lowest mortality rate was found for biliary tract perforations (12%), while the mortality of duodenal perforations was as high as 28%. The anatomical location of the perforation and the associated mortality rates are shown in Table 1.

We were able to show that the presence of bacteria resistant to Meropenem (27% vs. 13%; *p* = 0.003) and Tigecycline (30% vs. 14%; *p* = 0.008) and the combination Meropenem/Vancomycin (65% vs. 15%; *p* = 0.001) was associated with higher mortality. No significant impact on mortality was detected for resistance against other tested antibiotics (Table 6).

**Table 6:** Mortality related to biological sensitivity.

	Mortality rate				*p* value
	Cultured bacterias: sensitive		Cultured bacterias: resistant		
	%	n	%	n	
Ampicillin/Sulbactam	17	46/274	14	20/140	0.777
Piperacillin/Sulbactam	15	52/338	19	14/76	0.487
Meropenem	13	42/324	27	24/90	**0.003**
Cefuroxime	14	34/235	18	32/179	0.419
Cefotaxime	15	38/253	17	28/161	0.409
Ceftazidime	16	37/234	16	29/180	0.969
Tigecycline	14	51/363	30	15/51	**0.008**
Ciprofloxacin	17	37/221	15	29/193	0.502
Moxifloxacin	16	45/280	16	22/134	0.881
Vancomycin	15	22/149	17	44/265	0.575
Metronidazole	16	11/67	16	55/347	0.857
Cefuroxime/ Metronidazole	15	40/269	18	26/145	0.483
Cefotaxime/ Metronidazole	15	43/284	18	23/130	0.563
Meropenem/Vancomycin	15	61/408	65	4/6	**0.001**

Bold value indicates *p* < 0.05.

## Discussion

The aim of this study was to identify the microbes that are present in secondary peritonitis and to analyze their sensitivity to antibiotics commonly used in empiric treatment so as to evaluate appropriate treatment options.

The location of the origin of the peritonitis has an enormous impact on outcome, since the microbial flora differs markedly in different parts of the intestinal tract. Resistance of bacteria to antibiotics of second or last resort can influence mortality.

Secondary peritonitis is caused by hollow organ perforation or biliary infection and is the most common form of peritonitis, accounting for about 80% of all cases with peritonitis. However, improvements in intensive care and antimicrobial treatment as well as surgical technique have reduced the mortality of severe intra-abdominal infections with peritonitis from 90% in 1900 to between 10 and 25% (9, 11), which is in line with our results (mortality rate 15.9%). The pivotal role of surgical treatment for peritonitis is undisputed (5).

The strongest predictive factors for mortality are the degree of peritonitis, age and adequate source control (5). Furthermore, our study found age to be a significant risk factor for mortality, too (Table 1). The degree of organ dysfunction is assessed using the SOFA score. The mortality rate increases with the number of dysfunctional organs. In this study, we were also able to reveal a correlation between the SOFA score and mortality. Organ dysfunction due to septic disease in secondary peritonitis arises from a dysregulated response to the infection. The extent to which a specific spectrum of bacteria or other factors such as pre-existing diseases are also responsible for organ failure cannot be differentiated.

The mortality of intra-abdominal infections in our data varied, in regard to their origin. For intra-abdominal infections originating from the stomach and small intestine or colon, the mortality rate was found to be 15%, while perforations of the duodenum are associated with a mortality of 28%. These findings are in accordance to previously published data, but these studies rarely deal with the examination of microbes and the sensitivity of antibiotics.

Empirical antimicrobial therapy should be based on local epidemiology, individual patient risk factors for resistant pathogens, clinical severity of infection, and infection source^13^. In high risk patients, empiric treatment should be started using broad spectrum antibiotics, but the selection should not only be stratified by the severity of the peritonitis but also according to the localization of the perforated organ^14^.

Peritonitis is often a polymicrobial disease^11,15^. In earlier publications, the rate of *E. coli* was found to be 25%, while gram-positive cocci were found in over 30%^5^. This has changed within the last 15 years. Nowadays, in secondary peritonitis, gram-negative bacteria can be detected in approximately 60% of patients; more than 40% of these being *E. coli*, followed by Klebsiella pneumoniae. Gram-positive bacteria were found in 22% (most frequently *E. faecalis* and Streptococcus). In a previous study dealing with cholecystitis, gram-negative bacteria were found in 70% (32% *E. coli*), while gram-positive bacteria were detected in 24% (most commonly *E. faecalis* 8%; faecium 4%)^16^. In another large series, *E. coli* was found in 40% of severe peritonitis, followed by Streptococcus (29%), Enterococcus (8%), Klebsiella (7%) and Pseudomonas (7%)^17^. Comparable results were found in our trial with Enterococci in 20%, *E. coli* in 39% and Enterobacter species in 24% of the patients (Table 3). In addition, the frequent appearance of yeasts is often described^18–20^, but the clinical relevance is low.

Empiric antibiotic treatment for peritonitis should cover the habitual pathogens as well as less common bacteria^21^. The suggested regimes range from second or third generation Cephalosporin plus Metronidazole or Piperacillin/Sulbactam to Carbapenem or Tigecycline as single treatment or in combination with gycopeptide antibiotics (Vancomycin)^21^. In contrast, Klibanov et al. and Dupont et al. hypothesized that there is no difference between monotherapy and antimicrobial combinations^15,19^. Combining Cephalosporins with Metronidazole is promoted in recent empiric regimen guidelines^4,8,15,22^. Short high dosed antibiotic therapy with de-escalation in awareness of the bacterial flora is a recommendation of the antibiotic stewardship program to optimize the use of antibiotic medication^23,24^. There is little data on decision making for the selection of empiric treatment in secondary peritonitis^21^. The Cochrane Analysis published by Wong et al. reports comparable results of all tested antibiotic regimes in secondary peritonitis, in terms of clinical success; therefore, no recommendation for specific antibiotic regimes is given^11^.

According to our data, an empiric antibiotic therapy with Cephalosporin (second or third generation) in combination with Metronidazole has a low in vitro sensitivity rate between 55 and 73% (Table 5). Cephalosporin/Metronidazole is mainly effective against *E. coli*, Streptococci and Bacteriodaceae. We isolated *E. coli* in 39% of the examined cases, Streptococci in 8% and Bacteriodaceae in 22%. Cefuroxime or Cefotaxime with Metronidazole is effective against 65% and 69% of the germs found. Meropenem, on the other hand, is effective in 98% of the cases and covers the entire spectrum of germs investigated, so that in our opinion, this antibiotic should be preferred in critically ill patients with secondary peritonitis.

Meropenem and Imipenem are the most commonly used Carbapenems, which have sensitivity rates of over 90% for *E. coli* and Klebsiella pneumonia, which account for 50% of the detected microbes in secondary peritonitis^11,25,26^. Previously and also in our analysis, Meropenem showed excellent results for all bacteria except Enterococcus^17^. Overall, Meropenem reached sensibility results of 78% and in combination with Vancomycin even 98% for all locations. These data are in accordance with response rates reported in a previous trial (third generation Cephalosporin 72% and Carbapenem 98%)^20^. However, in recent years, clinicians have become dependent on Carbapenems for treating Extended Spectrum Beta-Lactamase (ESBL) infections, which emphasizes the importance of Carbapenem-preserving antimicrobial stewardship^13^. At the University Hospital, Hamburg-Eppendorf, the combination of meropenem and Vancomycin is administered as calculated treatment for patients in septic shock with onset of organ failure.

Moxifloxacin is sometimes used for treatment of intra-abdominal infections due to a previously reported response rate of 80%^26^, but in our study we found an in vitro sensibility rate of only 67%. Furthermore, extended use of Fluoroquinolones should be discouraged because of their selective pressure (mainly ESBL producing Entrobacteriaceae and MRSA)^13^. Tigecycline is effective in 88% overall. It shows excellent results for treatment of Enterococcus (> 99%)^25^, which is confirmed by our findings. A major problem, however, is the natural resistance of Enterococcus against various antibiotics. Correspondingly, presence of Enterococcus has been shown to be associated with increased mortality^27,28^. Enterococci were found in 10% of our patient population. Since Enterococci are resistant to many antibiotics and Meropenem is only of limited effectiveness, therapy with Tigecycline or Piperacillin/Sulbactam should be considered if Enterococci are revealed in the culture. In previous studies, Tigecycline in combination with Gentamicin or Ciprofloxacin was found to be effective against Enterobacteriaceae^29^.

Previously, it was shown that early empiric antibiotic treatment reduces morbidity and mortality in critically ill patients^4,19^. In multivariate analysis severe peritonitis, correct empiric antibacterial treatment and inadequate source control were independent prognosticators of mortality^16^. In contrast, in a multicenter trial, 39% of patients received inadequate initial empiric antimicrobial treatment. These patients showed significantly higher mortality (12% vs. 5%) as well as more surgical site infections (53% vs. 40%) compared to the patients receiving appropriate initial empiric therapy^30^ , while another trial found contradictory results with no significant impact on mortality^14^.

Antimicrobial treatment plays an important role in the management of peritonitis, but the rapid spread of multi-drug resistant bacteria like *E. faecium*, Methicillin-resistant Staphylococcus aureus (MRSA), Vancomycin resistant Enterococci (VRE) or Enterobacteriacae with Extended Spectrum Beta-Lactamase, has become a serious threat, especially in critical care medicine^14,16,30–32^.

It is known that ineffective antibiotic treatment increases the risk of antibiotic resistance three-fold^16^ and that early administration of broad-spectrum antibiotics reduces this risk^14^. To date, no consensus on type and duration of antibiotic treatment exists^16^. The duration of empiric treatment and even any antibiotic treatment remains frequently discussed. Studies have shown that the decrease of PCT levels (procalcitonin levels) seems to be a good marker to determine the end of antibiotic administration, while other authors suggest a duration of at least 5 days^21,33^.

The prevalence of invasive fungi has increased, but to date, the rate is low in secondary peritonitis^16^. Yeasts were detected in up to 22% of patients with severe peritonitis, but no association between their presence and mortality was found. Therefore, empiric coverage is not recommended^3^.

### Limitation of the trial

To increase the case load of our study we enrolled patients over a period of 10 years. This might include a recent change in microbial resistance patterns. Moreover, the methods of bacterial identification (Enterotube vs. MALDI (Matrix assisted laser desorption/ionization) tof) and resistance testing (agar diffusion vs. VITEK®) have evolved during the study period. In addition, significant changes in susceptibility interpretation rules (EUCAST) have been implemented, which may have led to systematic underestimation of the amount of in-vitro resistant bacteria for earlier cultures. In accordance to other publications, abdominal fluids were not cultured in all patients^3^. However, we report on a large cohort with analysis of microbial flora for different origins of secondary peritonitis and evaluation of their respective sensitivity rates.

## Conclusion

Besides early source control, appropriate empiric treatment plays a pivotal role in the treatment of secondary peritonitis. In this retrospective analysis we were able to show that the frequently used combination of Cephalosporin plus Metronidazole is not sufficient due to the biological resistance of the bacteria found. In critically ill patients, broad-spectrum antibiotics, such as Carbapenem plus Vancomycin with a sensitivity rate of 98% are recommended.
